# CD4^+^ T Cell Interstitial Migration Controlled by Fibronectin in the Inflamed Skin

**DOI:** 10.3389/fimmu.2020.01501

**Published:** 2020-07-24

**Authors:** Ninoshka R. J. Fernandes, Nicholas S. Reilly, Dillon C. Schrock, Denise C. Hocking, Patrick W. Oakes, Deborah J. Fowell

**Affiliations:** ^1^David H. Smith Center for Vaccine Biology and Immunology, Department of Microbiology and Immunology, University of Rochester Medical Center, Rochester, NY, United States; ^2^Department of Biomedical Engineering, University of Rochester, Rochester, NY, United States; ^3^Department of Physics and Astronomy, University of Rochester, Rochester, NY, United States; ^4^Department of Pharmacology and Physiology, University of Rochester Medical Center, Rochester, NY, United States; ^5^Department of Biology, University of Rochester, Rochester, NY, United States

**Keywords:** T cell, migration, extracellular matrix, fibronectin, inflammation, skin

## Abstract

The extracellular matrix (ECM) is extensively remodeled during inflammation providing essential guidance cues for immune cell migration and signals for cell activation and survival. There is increasing interest in the therapeutic targeting of ECM to mitigate chronic inflammatory diseases and enhance access to the tumor microenvironment. T cells utilize the ECM as a scaffold for interstitial migration, dependent on T cell expression of matrix-binding integrins α_V_β_1_/α_V_β_3_ and tissue display of the respective RGD-containing ligands. The specific ECM components that control T cell migration are unclear. Fibronectin (FN), a canonical RGD-containing matrix component, is heavily upregulated in inflamed tissues and *in vitro* can serve as a substrate for leukocyte migration. However, limited by lack of tools to intravitally visualize and manipulate FN, the specific role of FN in effector T cell migration *in vivo* is unknown. Here, we utilize fluorescently-tagged FN to probe for FN deposition, and intravital multiphoton microscopy to visualize T cell migration relative to FN in the inflamed ear dermis. Th1 cells were found to migrate along FN fibers, with T cells appearing to actively push or pull against flexible FN fibers. To determine the importance of T cell interactions with FN, we used a specific inhibitor of FN polymerization, pUR4. Intradermal delivery of pUR4 (but not the control peptide) to the inflamed skin resulted in a local reduction in FN deposition. We also saw a striking attenuation of Th1 effector T cell movement at the pUR4 injection site, suggesting FN plays a key role in T cell interstitial migration. In mechanistic studies, pUR4 incubation with FN *in vitro* resulted in enhanced tethering of T cells to FN matrix, limiting productive migration. *In vivo*, such tethering led to increased Th1 accumulation in the inflamed dermis. Enhanced Th1 accumulation exacerbated inflammation with increased Th1 activation and IFNγ cytokine production. Thus, our studies highlight the importance of ECM FN fibrils for T cell migration in inflamed tissues and suggest that manipulating local levels of ECM FN may prove beneficial in promoting T cell accumulation in tissues and enhancing local immunity to infection or cancer.

## Introduction

T cell recruitment to infected tissues is critical for pathogen clearance. Once T cells enter a site of inflammation, they need to scan the tissue to locate infection foci and to interact with antigen presenting cells (APCs) for reactivation and cytokine release (1). While much is known about leukocyte extravasation from blood vessels into tissues, the mechanisms that promote efficient T cell interstitial migration are poorly understood (2–5).

Parameters such as tissue confinement, display of chemotactic factors and the composition of the ECM all impact the ability of leukocytes to traverse the 3D space (6–8). The use of intravital multiphoton microscopy (IV-MPM) of inflamed and infected tissues has provided important insight into how leukocytes navigate the dense three-dimensional microenvironments of non-lymphoid tissues (9–14). In many tissues (9, 10, 15, 16), T cell interstitial migration has been shown to be non-directional or random raising questions as to the guidance cues that facilitate T cell localization to infection foci. Studies in the brain and skin observed T cells crawling along fibrillar structures (9, 13) suggesting T cells utilize the ECM as a scaffold for interstitial migration. The ECM is extensively remodeled in inflamed tissues with alterations in matrix density and composition (7, 17) likely impacting the mechanism of T cell motility (18). In the inflamed dermis, changes in ECM density correlated with a requirement for matrix-binding integrins for T cell interstitial migration (9). Specifically, the blockade of RGD-binding integrins α_V_β_1_ or α_V_β_3_ resulted in Th1 effector T cell arrest (9). Therefore, matrix components that contain an RGD-sequence (fibronectin (FN), vitronectin, osteopontin, thrombospondin, tenascin-C) may facilitate T cell tissue scanning. In contrast, a number of cancer studies suggest the ECM can function as a barrier to intra-tumoral T cell migration (19–21).

Enhanced deposition of FN has been observed in both acute and chronic inflammatory settings and may function as an important substrate in T cell integrin-dependent interstitial migration (9). A number of studies of fibrosis have targeted FN to block overt ECM deposition and limit tissue pathology (22–25). These models take advantage of naturally occurring bacterial adhesins that are known to bind to FN and facilitate microbial cell attachment and host cell infection (26–28). pUR4, or FUD, is a polypeptide based on the F1 adhesion of *Streptococcus pyogenes* and is a specific inhibitor of FN matrix deposition by blocking the FN N-terminus cell binding sites required for cell-mediated FN fibril assembly (29, 30). In fibrotic models, FN deposition was attenuated and inflammation reduced by pUR4-treatment (22–25). Here, we use pUR4 as a tool to address the requirement for matrix FN in T cell motility and to test the efficacy of targeting FN to manipulate T cell-meditated immunity.

Using IV-MPM, we show that T cells migrate along flexible FN fibers, often deforming the fibers as they migrate along the ECM scaffold. Blockade of FN deposition by pUR4 treatment inhibited T cell interstitial migration resulting in a marked perivascular T cell accumulation. Despite limiting the availability of FN as a substrate for T cell migration, our studies show pUR4 treatment also enhanced T cell adhesion; possibly through promoting a conformational change in the integrin-binding domain to alter adhesion dynamics (31–33). Thus, pUR4 treatment led to enhanced Th1 accumulation at the treatment site. The accumulated T cells in the tissue following pUR4 treatment were fully activated with enhanced IFNγ production. Thus, pUR4 treatment appears to locally exacerbate inflammation in acute T cell-mediated responses. This alternative mode of action may be detrimental in chronic inflammation such as autoimmunity but may represent a novel way to increase T cell function in tumors or at sites of chronic infection.

## Materials and Methods

### Mice

Wild-type (WT) BALB/c mice were from the National Cancer Institute. DO11.10 TCR Tg+ mice (Jackson Laboratories) were crossed to BALB/c Thy1.1^+^ mice and/or Kaede Tg^+^ mice (34). All mice were maintained in a pathogen-free facility at the University of Rochester Medical Center. All mouse procedures were performed with approval of the University of Rochester's Institutional Animal Care and Use Committee.

### T Cell Culture and Adoptive Transfers

For *in vitro* effector T cell priming, CD4^+^ cells were enriched from lymph nodes and spleens as previously described (35) and naïve T cells selected on a CD62L MACS column (Miltenyi). T cell-depleted splenocytes were irradiated (25Gy) as APC. 3 × 10^5^ naive T cells were stimulated with 1.2 × 10^6^ APC, 1μM ovalbumin (OVA) peptide, IL-2 (10 U/ml), IL-12 (20 ng/ml) and anti-IL-4 (40 μg/ml; 11B11) for Th1 skewing and cultured for 5 days. After 5 days of culture, Th1 cells were washed, counted and labeled with CellTracker Orange (CMTMR, Invitrogen) or isolated from GFP-Kaede transgenic mice for fluorescent detection (34). Th1 cells (7.5 × 10^6^) were adoptively transferred into mice i.v. prior to immunization.

### Purification of pUR4 and III-11C Peptide

pUR4 and III-11C polypeptides were expressed in bacteria with a His-tag for Nickel-NTA resin column purification as previously described (23). pUR4 binds to the amino-terminus of FN and blocks FN matrix assembly (29, 36). III-11C control peptide is a terminal fragment (68-mer) of FN III-11C module (23). Endotoxin levels were quantified using Pyrogene Recombinant Factor C endotoxin detection assay (Lonza) and removed with an Acrodisc filter with Mustang E membrane (Pall laboratory).

### Dermal Inflammation and Peptide Treatment

Mice were immunized intradermally (i.d.) in the ear pinna with 1 μg of OVA or Keyhole Limpet Haempcyanin (KLH) protein emulsified in Complete Freund's Adjuvant (CFA). Seven-hundred micro molar pUR4 or III-11C peptide (50 μg per injection) was injected i.d. 1 day prior and 2 days after immunization.

### Intravital Multiphoton Imaging

Mice were imaged as previously described (9, 37) on an Olympus Fluoview FVMPE-RS twin-laser MPM. Mice were anesthetized with isoflurane and maintained at 2% in room air with an isoflurane vaporizer-ventilation machine (Kent VetFlo). Once mice were anesthetized, the ventral side of the ear was affixed to the coverslip on a custom-built platform for imaging as described in previous studies (9, 37). The microscope objective was heated to 37°C degrees to maintain dermal temperature in the ear during the imaging session. Mouse body temperature was also maintained at 37°C degrees using a heated water pad (Kent Scientific) and a heating block (WPI). Images were acquired using Olympus Fluoview FVMPE-RS twin-laser multiphoton microscope system with two lasers—spectra-physics InsightX3 laser (range of wavelengths: 690–1,300 nm) and spectra-physics MaiTai HP DeepSee Ti:Sapphire laser (range of wavelengths: 690–1,030 nm) with DM690-870 and DM690-1050 coupling mirrors. An Olympus 25x objective (numerical aperture, 1.05) was used to collect fluorescence for deep-tissue multiphoton imaging and the signal was detected with four proprietary photomultipliers. A MaiTai laser was tuned to 800 nm for excitation of fluorophores AF647 and phycoerythrin (PE), and InsightX3 to 900 nm for excitation of Kaede and SHG. To visualize fibronectin, AF488 was excited at 985 nm. The emission of blue (SHG), green (Kaede or AF488), near-red (PE), and far-red (AF647) was detected with a proprietary filter set (Olympus Fluoview FVMPE-RS). For time series analyses, 60 μm z-stacks of 512 × 512-pixel images were captured every minute with a z step-size of 4 μm.

Blood vessels were visualized by intra-venous (i.v.) injection of fluorescently-conjugated anti-CD31 Ab (clone 390) immediately prior to imaging; APCs visualized by i.d. injection of 1 μg CD11c-PE Ab (clone N418) and Fc block, anti-CD16/32 Ab (clone 2.4G2) 2 h prior to imaging. Purified human plasma FN and control N-ethylmaleimide (NEM)-treated plasma FN were generated and conjugated to Alexa Fluor 488 (AF488) as described (38) and 100 μg injected i.v. 4 h prior to imaging.

### Image Analysis

Image analysis was done in Volocity (Perkin Elmer) or Imaris (Bitplane). Motility was analyzed for 20–30 min and T:APC contacts for 50–60 min. Mean squared displacement (MSD) calculated using $\langle {\left(x-x_{0}\right)}^{2}\rangle =\sum_{n=1}^{N}{\left(x_{n}(t)-x_{n}(0)\right)}^{2}$, where N is the number of cells to be averaged, *x*_*n*_(0) is the reference position of each cell, and *x*_*n*_(*t*) is the position of each cell at time t. Motility coefficient for each cell track was calculated in MATLAB to determine the slope of the best-fit linear regression for the squared displacement and time measurements for the first 10 min with a coefficient of determination (*r*^2^) >0.8. For cell distance from ECM-fibers (FN or SHG) or CD31^+^ vessels, fibers or vessels and cells were volumetrically rendered in 3D using Imaris. Distance transformation Xtension was applied to the fiber or vessel and to the T cell surface, resulting in a new channel with the intensities equal to the distance from the surface object. Minimal distance of each cell from the closest fiber or vessel, was calculated using Imaris. For T:APC contacts, 3D surfaces for CD11c+ cells and T cells were generated using the Imaris surface tool. The 3D volumetric overlap between the two surfaces was identified as a contact and measured over time.

For fiber alignment, the local orientation was determined by calculating local moments based on the image intensity as previously described (39). Briefly, the image was broken down into a series of small windows ~33 μm by 33 μm that overlapped by 50%. Each sub window was filtered into periodic and smooth components (40) and the 2D Fast Fourier Transform (FFT) was calculated. A circular mask the size of the image sub window was applied and the central image moments were calculated using the intensities from the FFT. The orientation of the FFT image was determined by calculating the eigenvectors for the covariance matrix of the central image moments. The orientation of the original sub-window is related by a 90° rotation from the orientation of the FFT image. This process was then repeated with each sub window of the image to create a vector field of local orientations for the whole image. This process was repeated for each plane and channel of a multiplane image stack. To compare vector distributions the average order parameter, defined as cos^2^ 〈θ^2^〉 with θ the angle between two vectors, was calculated. Two control distributions were created for statistical comparisons. First, we created a randomized version of the collagen orientations. This distribution contained all the same vectors as the collagen distribution, but assigned to random positions in the vector field. Second, we created a completely random distribution of vectors. These vector fields were then compared to the fibronectin orientations as control distributions.

### *In vitro* T Cell Migration

Th1 migration was assessed under confinement, using an under-agar model as described (41). Glass coverslips (EMS) were coated with 50 μg/mL FN (Millipore) for 60 min at RT. For pre-treatment of FN, 200 nM FN was incubated with 500 nM pUR4 or III-11C for 30 min at RT before coating coverslips. The coverslip was overlayed with 0.5% agar in serum-free RPMI and 10 ng/mL CXCL10. A small agar plug was then removed to allow ~1 × 10^5^ Th1 cells to be added to the coverslip and slides incubated for 30 min at 37°C to allow for cells to migrate under the agar. For pre-treatment of T cells, 1 × 10^5^ Th1 cells were incubated with 500 nM pUR4 or III-11C for 30 min at 37°C in serum-free RPMI and washed before adding to the imaging chamber. Cells were imaged at 30 s intervals for 20 min using a 20x Plan Fluor oil immersion lens (NA = 0.75) on an inverted motorized Ti-E microscope (Nikon) using DragonFly spinning disk confocal system (Andor).

### Flow Cytometry

Cells were harvested from the ear and stained with fixable live/dead cell stain (ThermoFisher) (42). For *ex vivo* intracellular staining of cytokines, brefeldin A (1 μg/ml) was added to all media and wash buffers to block cytokine secretion (43). Leukocytes were stained with Abs (Biolegend) to CD45 (30-F11), CD4 (RM4-5) and Thy1.1 (H1S51), fixed with cytofix/cytoperm (BD Biosciences) and stained with anti-IFNγ Ab (XMG1.2). Analyzed by FACS using the BD LSR II.

### Immunohistochemistry (IHC)

Frozen sections (8 μm) of ear tissue were fixed with acetone prior to IHC. Tissues were blocked with 1% newborn mouse serum and 10 ng/ml Fc block (anti-CD16/32 Ab). Antibodies used were as follows: anti-FN (96-23750; Abcam), anti-type III collagen (1330-01; Southern Biotech), anti-rabbit Ig (A31573; ThermoFisher), anti-goat Ig (Jackson Immunoresearch). The sections were imaged using 20X and 100X oil objectives on FV1000 Olympus laser scanning confocal microscope. For FN detection following pUR4 injection, 4 μm thick sections from 10% formalin and paraffin embedded ears were stained with anti-FN Ab (96-23759, Abcam) and biotinylated anti-rabbit Ig (Vector Labs). Biotinylated Abs were detected with streptavidin-HRP and 3,3'-Diaminobenzidine. Sections were counterstained with hematoxylin and visualized using a Leica color camera microscope.

### Statistical Analyses

GraphPad Prism software was used for all statistical analysis. For motility parameters, non-parametric Mann-Whitney tests were applied. For cell frequency and number observations, one-way ANOVA with Tukey's multiple comparisons were conducted. For all other analyses, two-tailed ANOVA tests with Sidak's multiple comparison test were performed. Data were reported as mean ± standard error of the mean (SEM). ^*^= *p* < 0.05, ^**^= *p* < 0.01, ^***^ = *p* < 0.001, ^****^= *p* < 0.0001.

### Data Sharing Statement

For original data, please contact Deborah_fowell@urmc.rochester.edu.

## Results

### Association of Migrating T Cells With FN Fibers in the Inflamed Dermis

**Figure 1:**
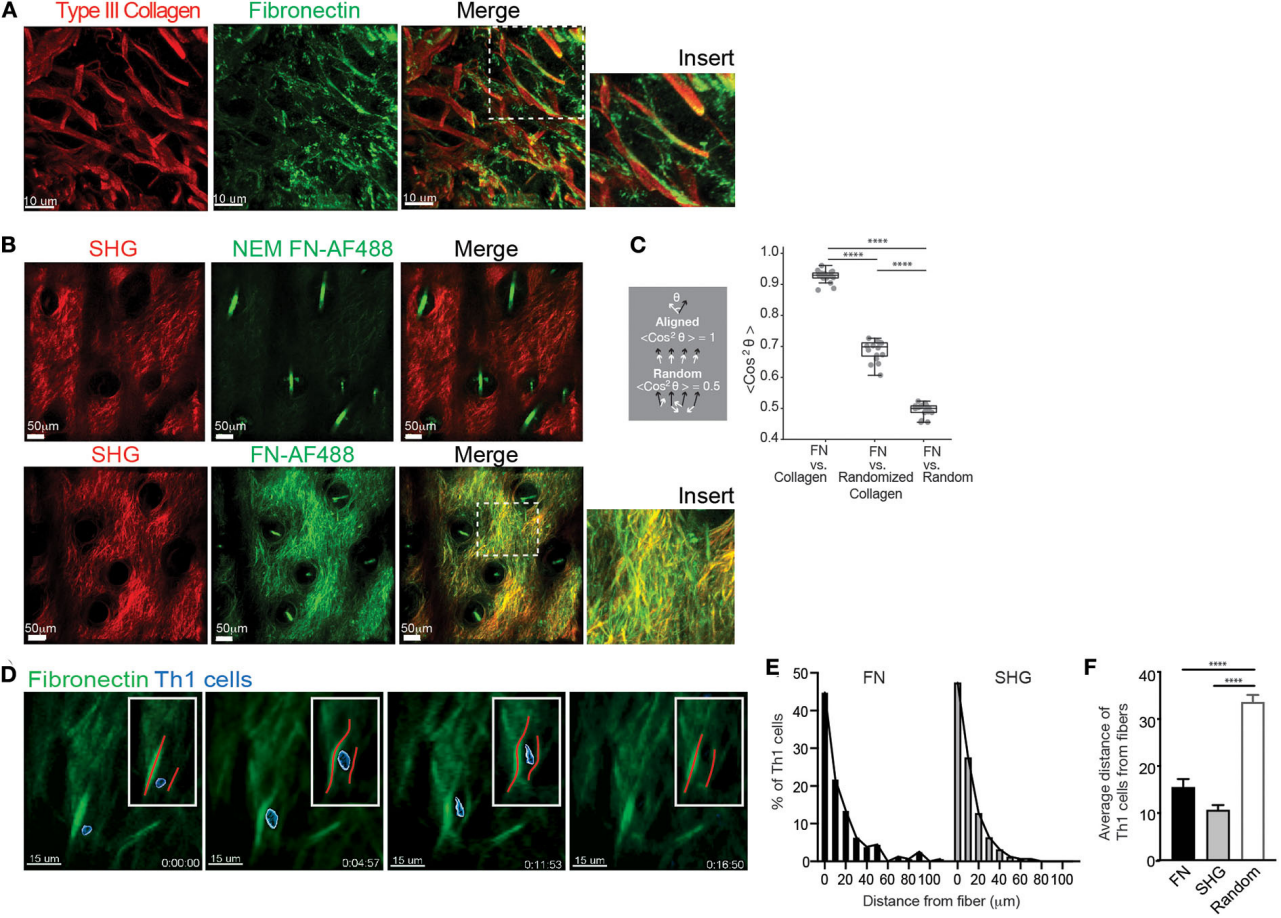
FN fibrils and Th1 migration. WT mice were intradermally immunized with OVA/CFA in the ear pinna and assessed by IHC of frozen sections **(A)** or IV-MPM **(B)** 3 days post-immunization. **(A)** The ear tissue was excised and frozen for IHC. Eight micrometer slices were imaged using confocal microscopy. Type III collagen (red) and FN (green), scale bar 10 μm. **(B)** 100 μg of control NEM-treated FN-AF488 (NEM FN-AF488) (top) or FN-AF488 (bottom) were given i.v. to immunized mice 4 h prior to IV-MPM. Second harmonic generation (SHG) (red), AF-488 FN (green), scale bar 50 μm. **(C)** Measurement of the alignment of the ECM images in **(B)** measured as the average cosine squared of the angle between the local fibronectin orientation and either the local collagen orientation, a randomized distribution of the collagen orientations, or a random distribution. Two perfectly aligned distributions have a value of 1, while two completely random distributions have an average value of 0.5. Each dot represents one z-plane of the image stack, containing 200+ vectors. Statistics by Mann-Whitney test. ****p < 0.0001. **(D)** OVA-specific Th1 cells labeled with CMTMR were adoptively transferred to WT mice and recipient mice immunized with OVA/CFA. Day 3 post-immunization, mice received 100 μg FN-AF488 4 h prior to IV-MPM. Th1 cells (blue, pseudo-colored), FN-AF488 (green), scale bar 10 μm. Inset, red lines placed along the midline of the FN fiber. Time lapse sequence of images from Video 1. **(E,F)** Quantitation of Th1 distance to nearest fiber, either FN or SHG. **(E)** Frequency distribution of Th1 cells from FN and SHG fibers. **(F)** Th1 cell distance from FN or SHG fibers compared to random distribution in same imaging field (Random). Number of cells imaged, >150. Statistics by ANOVA. ****p < 0.0001. Two to three independent experiments.

Our previous studies utilizing IV-MPM have shown that Th1 cells migrate along SHG fibers (collagen) in the inflamed dermis utilizing α_V_β_1_/α_V_β_3_ integrins and RGD-containing ligands (9). Given the α_V_β_1_/α_V_β_3_ integrins do not bind to collagen itself, it was presumed that other RGD-containing ECM ligands were physically associated with the collagen fibers. We first used confocal imaging to determine the spatial relationship between collagen fibers and the canonical-RGD-containing ECM component, FN, in the inflamed dermis (Figure 1A). Mice were immunized in the ear pinna with a protein antigen, OVA, emulsified in Complete Freund's Adjuvant (CFA, OVA/CFA) and FN localization examined by IHC of frozen skin sections day 3 post-immunization. FN was distributed along many of the collagen fibers and, itself, formed fibers in the interstitial space between the collagen fibers (Figure 1A).

The SHG signal generated with multiphoton microscopy had been used as a surrogate for fibrillar structures in tissues [type I and III collagen in the skin (9)] and has enabled the intravital study of T cell movement relative to the tissue ECM architecture. However, the specific ECM ligands that Th1 cells interact with have not been visualized *in situ*, in real time. To detect FN during IV-MPM imaging, we used Alexa Fluor 488-conjugated plasma FN (FN-AF488) that is rapidly assembled into interstitial ECM fibers (38, 44). To confirm the visualization of the ECM-form of FN specifically, plasma FN was treated with N-ethylmaleimide (NEM) prior to labeling (NEM FN-AF488) preventing its assembly into the ECM (44). FN-AF488 or NEM FN-AF488 was injected i.v. into OVA/CFA immunized mice 4 h prior to IV-MPM of the inflamed dermis. No signal was detected from the control NEM FN-AF488 (Figure 1B) but FN-AF488 was readily detected along with the SHG fibers in the inflamed dermis (Figure 1B). FN-AF488 fibers occurred coincident with SHG and as distinct FN fibers, similar to the confocal images. As both the fibronectin and collagen (SHG) networks are dense 3D networks, we would expect a substantial amount of co-localization of the FN-AF488 and SHG signal independent of association between the two ECM proteins. To quantify similarities between these networks, we therefore used the fluorescent signal to determine the local alignment of each ECM protein and measured the difference between the orientations of the fibronectin and collagen matrices (Figure S1). We found that the FN and Collagen networks were almost perfectly aligned, consistent with considering them a single network (Figure 1C). As controls, we compared the fibronectin orientations with a randomized distribution of the collagen orientations and a completely random distribution (Figure S1). In both cases, the collagen network was significantly more aligned with the fibronectin network than with either control distribution (Figure 1C).

To determine the positioning of migrating T cells in relation to the FN fibers, we i.v. transferred CMTMR-labeled *in vitro*-generated OVA-specific DO11.10 TCR Tg^+^ Th1 cells into mice and immunized with OVA/CFA. Mice were imaged d3 post-immunization, as previously described (9), with FN-AF488 injected i.v. 4 h prior to imaging the inflamed dermis by IV-MPM. Th1 cells migrated in close association with the FN fibers (Figure 1D, Video 1, Figure S1). Quantitative analysis of the position of Th1 cells relative to the ECM fibers showed that the distribution of Th1 cells relative to FN or SHG fibers was similar (Figure 1E), with the majority of cells being <10 μm from a fiber. As a control, we compared the distance of Th1 cells from FN or SHG fibers with a randomized distribution of cells and found that Th1 cells were positioned significantly closer to both FN or SHG than if randomly distributed within the same tissue field (Figure 1F). Real-time imaging revealed flexibility in the FN fibers, similar to that seen in *in vitro* collagen matrices (45), with a temporary deformation of the FN network coincident with migrating Th1 cells (Figure 1C, Video 1). Thus, Th1 cell movement appears to result in temporary changes to the matrix, consistent with the idea that T cells may “push” or “pull” against the FN fibers during interstitial migration (45).

### Local FN-Inhibition Attenuates Th1 Cell Interstitial Migration in the Inflamed Dermis

**Figure 2:**
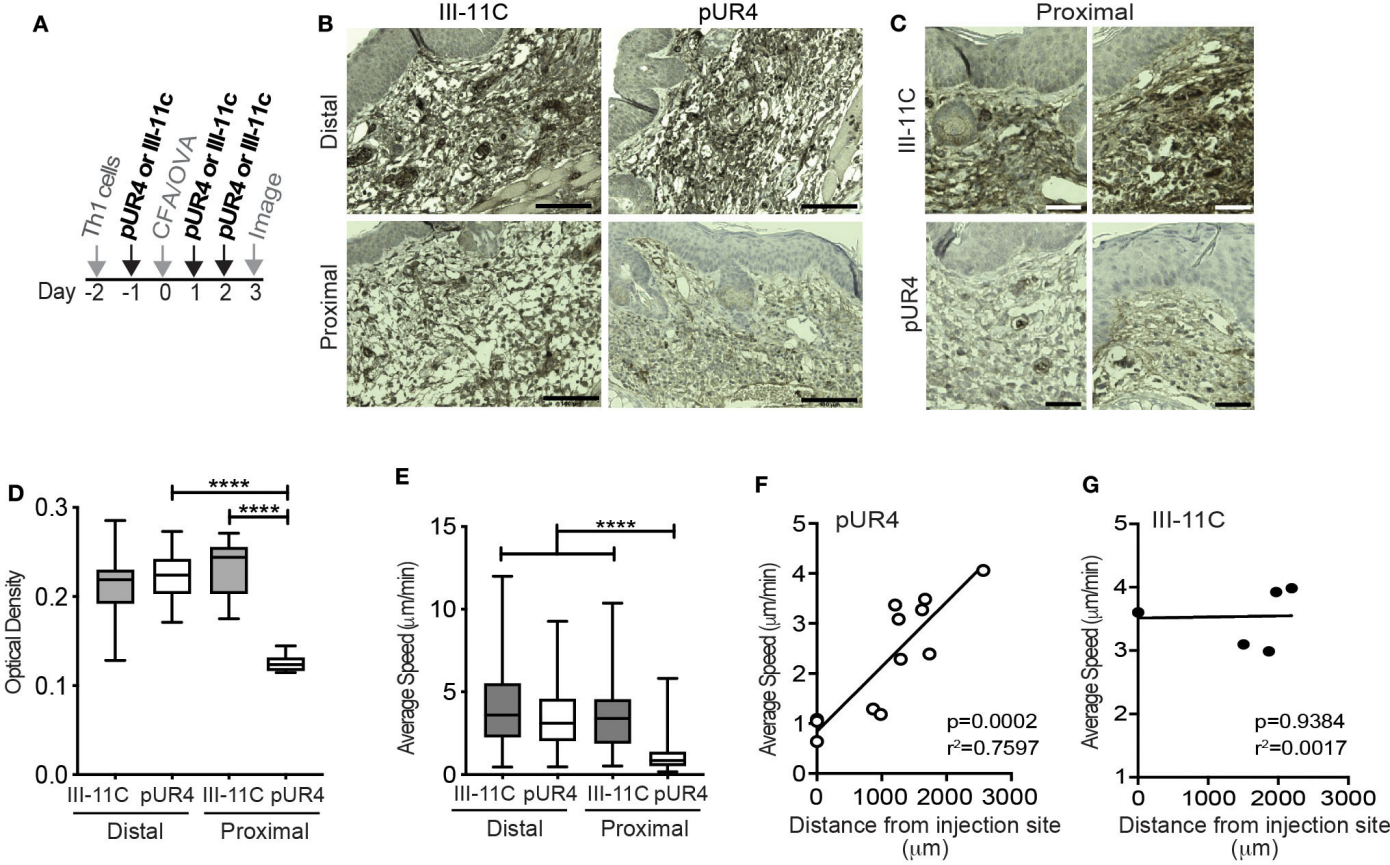
Local dermal pUR4 treatment blocks Th1 cell interstitial motility. *In vitro*-generated OVA-specific Kaede^+^ (green) Th1 cells were adoptively transferred to mice prior to intradermal administration of pUR4 or the control polypeptide III-11C in the ear pinna and immunization with OVA/CFA. **(A)** Treatment scheme, pUR4 or III-11C were administered day−1 and days 1 and 2 following immunization. Adoptively transferred Th1 cells were imaged by IV-MPM in the pUR4 or III-11C-treated dermis day 3-post OVA/CFA immunization. **(B,C)** IHC of paraffin-embedded skin sections from mice treated with pUR4 or III-11C, day 3 post-immunization. FN (brown), hematoxylin (blue), scale bar 100 μm. **(C)** Representative images from two independent experiments proximal to the injection site of the pUR4 and III-11C treated dermis, scale bar 50 μm. **(D)** Quantitation of FN staining by pixel density analysis in Fiji, from **(B)**. **(E)** Average speed of Th1 cells proximal or distal to the pUR4/III-11C injection sites. **(F,G)** Correlation between average speed of Th1 cells and distance away from the pUR4 **(F)** and III-11C **(G)** injection sites. Each symbol, mean speed of all cells in that imaging volume. **(D)** Statistics by one-way ANOVA. **(E)** Statistics by Kruskal-Wallis with Dunn's multiple comparisons test. **(F,G)** correlation was assessed using a standard two-tailed *t*-test. ****p < 0.0001. At least three independent experiments.

To test if FN is an important ECM ligand for T cell interstitial migration, we inhibited FN deposition in the skin using the polypeptide pUR4 (or FUD), a polypeptide based on a bacterial adhesin that specifically inhibits FN matrix deposition (22–25). pUR4, or control polypeptide III-11C, was administered intradermally in the mouse ear pinna one day before immunization (d-1) and day 1 and day 2 (d1 and d2) post OVA/CFA immunization (Figure 2A). Using IHC to determine FN expression in the inflamed dermis, we observed a significant decrease in FN staining proximal to the pUR4 injection site (Figures 2B,C and quantified in Figure 2D). Therefore, intradermal pUR4 treatment locally impacted FN availability. We next examined Th1 cell migration in the OVA/CFA immunized dermis relative to the pUR4 dermal injection site (Figures 2E–G). Th1 motility was significantly reduced proximal to the pUR4 injection (approx. <1,000 μm) (Figure 2E) but not at a distal site in the same ear pinna (Video 2), compared to III-11C injection (Video 3). Plotting average speed against imaging distance from the injection site (Figures 2F,G), we observed a positive correlation between the average speed of cells and the distance of our imaging volume from the pUR4 injection site (Figure 2F) but not the III-11C control (Figure 2G).

**Figure 3:**
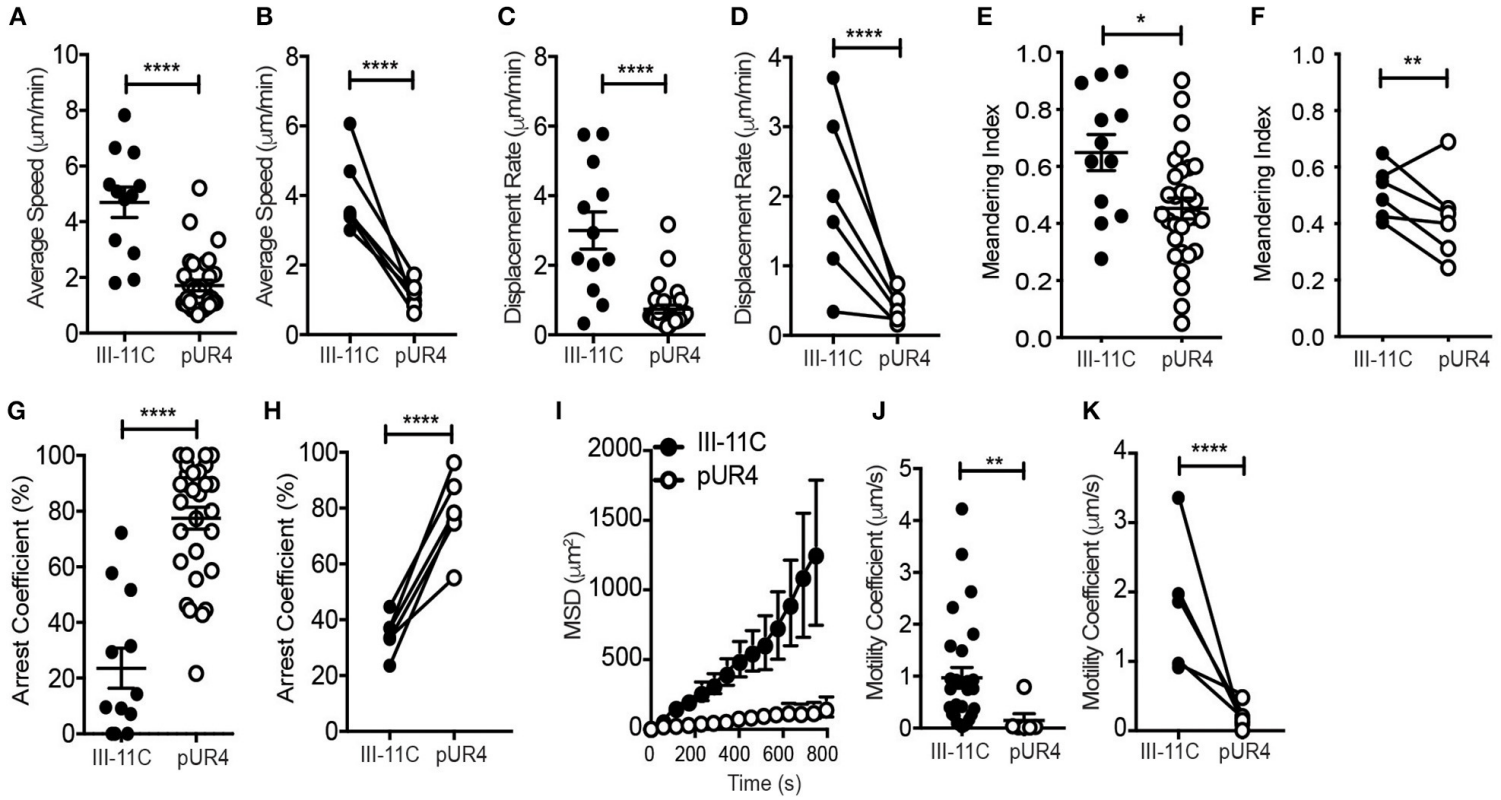
Intradermal dynamics of Th1 cell interstitial migration following pUR4 treatment. Th1 transfer, OVA/CFA immunization and pUR4-treatement as in Figure 2A. T cell migration assessed by IV-MPM day 3 post-immunization. **(A)** Average T cell speed per cell over a 20–30 min imaging time; representative imaging volume, each symbol an individual cell. **(B)** Average speed per mouse across multiple experiments, pUR4 and III-11C treatment paired by experiment. **(C,D)** Displacement rate and **(E,F)** Meandering index. **(C,E)** Individual cells in representative experiment. **(D,F)** Average per mouse across experiments. **(G,H)** Th1 arrest coefficient. **(G)** Individual cells in representative experiment. **(H)** Average per mouse across experiments. **(I)** Mean squared displacement (MSD) for Th1 cells in pUR4 or III-11C treated OVA/CFA inflamed dermis. **(J,K)** Motility coefficient of Th1 cells in pUR4 or III-11C treated OVA/CFA inflamed dermis. **(J)** Individual cells in representative experiment. **(K)** Average per mouse across experiments. Average speed and displacement rate were analyzed using Volocity. Arrest coefficient, MSD, and motility coefficient analysis was calculated using custom scripts written in MATLAB. Statistics were performed using Mann-Whitney test **(A,C,E,G,J)** and paired *t*-test **(B,D,F,H,K)**. ****p < 0.0001, **p < 0.01 and *p < 0.05. At least three independent experiments.

Further analysis of the motility dynamics of transferred Th1 cells in the inflamed dermis revealed a striking inhibition on T cell migration at the pUR4-treated site (Figure 3). Average speed and displacement rate (displacement of the cell from its initial position to its final position over the total time of the entire cell track) of Th1 cells in the pUR4-treated inflamed dermis was significantly reduced compared to the control III-11C-treated ear (Figures 3A–D). Similarly, the meandering index, a measure of the confinement of the track (displacement over the distance traveled), was also significantly decreased in the pUR4-treated inflamed dermis (Figures 3E,F). Data are shown for individual cells in a single experiment (Figures 3A,C,E) and the average values across multiple experiments, paired for mice in the same experiment (Figures 3B,D,F). Moreover, the arrest coefficient, which is a measure of the proportion of time a cell spends arrested (i.e., has an instantaneous velocity of <2 μm/min) (46), was significantly increased in the pUR4-treated inflamed dermis (Figures 3G,H) suggesting many of the cells were non-motile. Indeed, only 33% of Th1 cells had an average speed >2 μm/min in the pUR4-treated ear compared to 83% in the control-treated inflamed ear (Figure 3A). This pattern of movement suggests that FN manipulation impacts the ability of CD4+ effector Th1 cells to scan the tissue effectively. To quantify this, we analyzed cell tracks using two measures of exploration: the mean squared displacement (MSD) and motility coefficient (Figures 3I–K). MSD (the average of the squared displacement of all cell tracks over time) is a measure of the relative amount of tissue explored. While the motility coefficient (the slope of the squared displacement of each cell track) is a measure of the likelihood that a cell moves away from its point of origin. Both the MSD (Figure 3I) and motility coefficient (Figures 3J,K) were significantly decreased following pUR4 treatment. Thus, treatment with the FN inhibitor, pUR4, decreases T cell motility within the inflamed dermis and limits tissue exploration.

### pUR4 Can Act on FN to Tether Th1 Cells and Limit Migration

**Figure 4:**
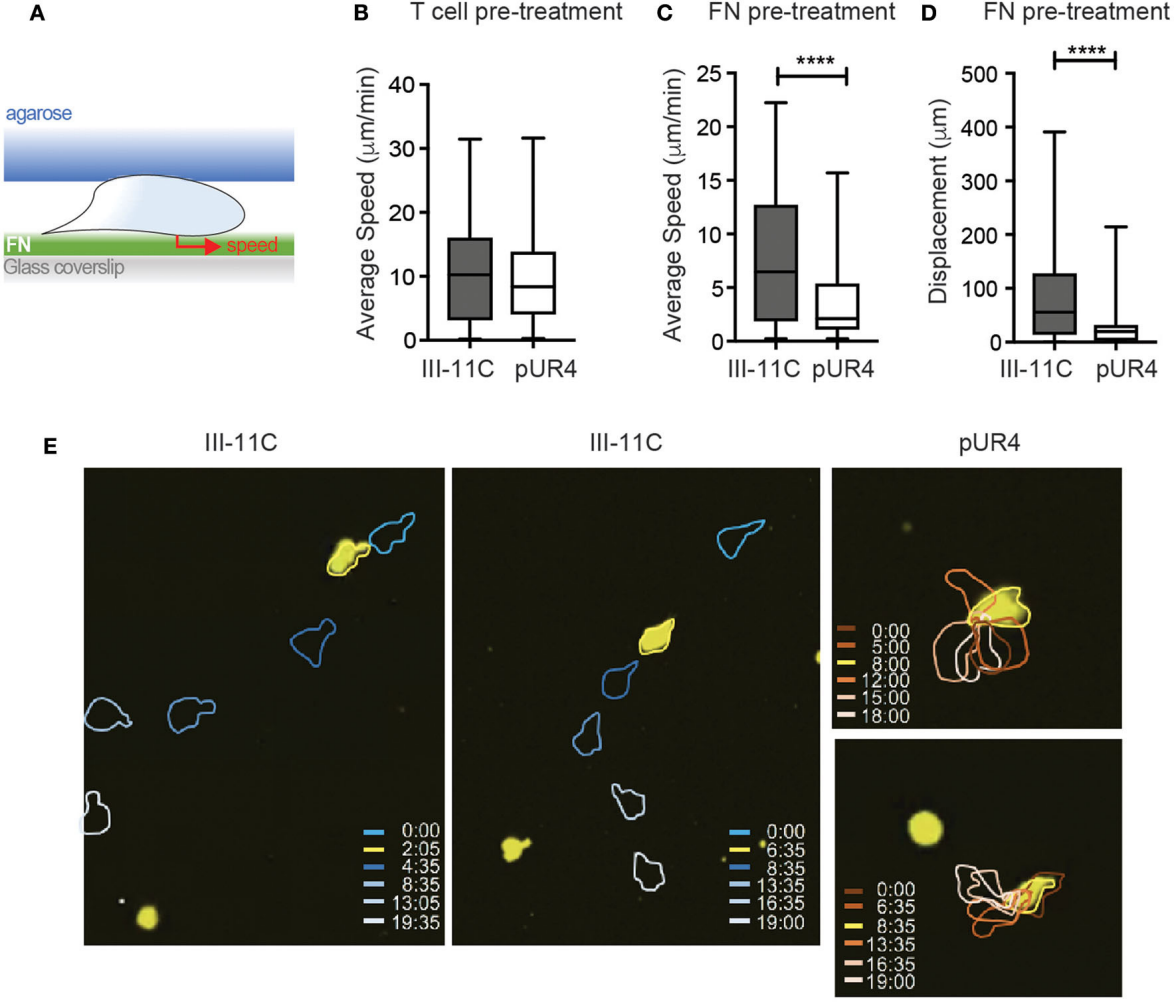
pUR4-treated FN halts Th1 migration *in vitro*. **(A)** Schematic of *in vitro* Th1 migration under confinement. **(B)** Pre-treatment of Th1 cells with pUR4 or III-11C before loading under agar on FN coated coverslip, average speed per cell. **(C,D)** Pre-treatment of FN with pUR4 or III-11C before coating coverslip. **(C)** Average speed of Th1 cells under-agar on the different FN treated surfaces. **(D)** Displacement of Th1 cells on the different FN treated surfaces. Statistics by Mann Whitney, ****p < 0.0001. **(E)** Sample time-lapse sequences for Th1 cells (yellow) on III-11C pre-treated FN (left, center) and pUR4 pre-treated FN (right), color-coded time scale in mins. Two to four independent experiments, 300–400 cells per group per experiment (see Videos 4, 5).

Lack of T cell migration in the presence of pUR4 could be due to too little or too much adhesion (47). Cells could arrest due to the absence of sufficient FN to gain traction or due to the ability of pUR4/FUD to induce a conformational change in FN that alters integrin-binding avidity (31, 32) leading to enhanced adhesion. Moreover, we cannot rule out additional effects of pUR4 acting directly on the T cells (in the absence of FN) to induce aberrant signaling for migration. To begin to assess mechanisms of action of pUR4, we developed an *in vitro* T cell migration system, based on previous models of T cell movement under confinement (41). Th1 cells were confined on FN-coated glass coverslips under agarose (Figure 4A) and migration imaged in real time. To test whether pUR4 attenuated T cell migration directly, Th1 cells were preincubated with pUR4 or III-11C, washed and then loaded into the FN-coated chamber. Th1 cells migrated vigorously (average 10 μm/min) on FN and there was no evidence that T cell pre-treatment with pUR4 altered T cell migration (Figure 4B, Video 4). To determine if pUR4 alters FN:integrin avidity, FN was pre-incubated with pUR4 or III-11C before coating the glass coverslips. pUR4 did not alter the amount of FN that bound to the glass coverslip (Figure S2), but did have a marked effect on FN's ability to support Th1 movement (Figures 4C–E, Video 5). pUR4-pretreatment of FN reduced the average speed of Th1 cells and markedly attenuated their displacement (Figures 4C,D, Figure S3). As observed in the movie (Video 5) and the sample time-lapse sequence(s) (Figure 4E), Th1 cells appeared tethered to the pUR4-treated FN substrate. These data are consistent with the idea that pUR4 may modify FN to enhance T cell adhesion, through a conformational change in FN that increases integrin binding (33).

### Perivascular Th1 Cell Accumulation

**Figure 5:**
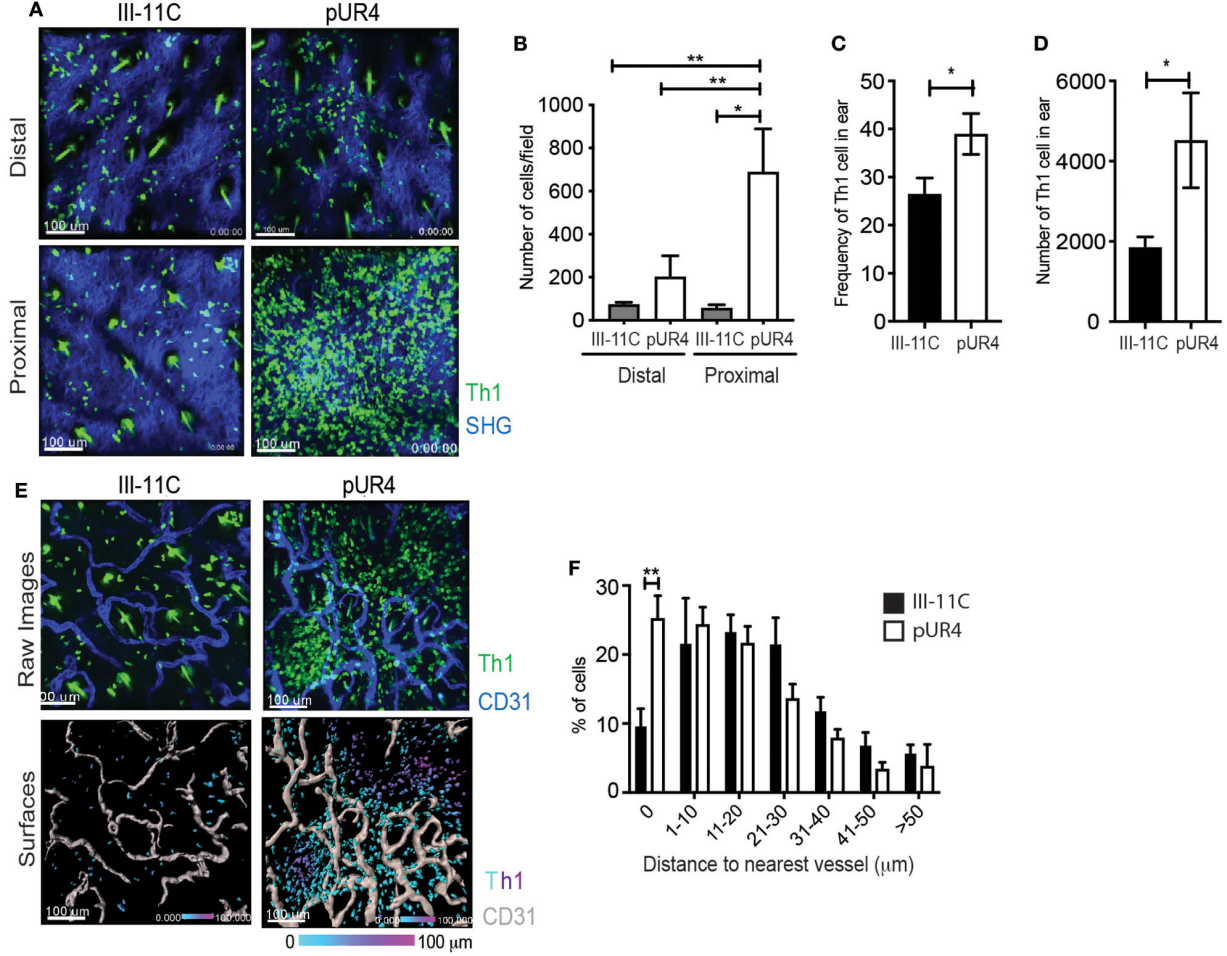
Perivascular Th1 cell accumulation in the pUR4-treated inflamed dermis. Adoptively transferred Kaede^+^ Th1 cells were imaged by IV-MPM in the pUR4 or III-11C-treated dermis day 3-post OVA/CFA immunization. **(A)** Representative images of Th1 cells accumulation proximal and distal to the pUR4 or III-11C injection site. Th1 cells (green), SHG (blue), scale bar 100 μm. **(B)** Quantitation of the number of Th1 cells in the imaging field, statistics by ANOVA. **(C,D)** Enumeration of Thy1.1^+^ transferred Th1 cells by Flow cytometry. Frequency of Th1 cells within the CD4^+^ population **(C)** and number **(D)** of Thy1.1^+^ cells in the pUR4 or III-11C-treated dermis day 3-post OVA/CFA immunization, statistics by unpaired *t*-test. **(E)** Representative images of Th1 cell position relative to CD31^+^ blood vessels, proximal to the pUR4 or III-11C injection site. AF647-labeled CD31 Ab administered i.v. immediately prior to imaging. Top, raw images; Th1 cells (green), SHG (blue), scale bar 100 μm. Bottom, 3D surfaces of vessels (gray) generated in Imaris and cells color coded based on distance to the nearest vessel. **(F)** Quantitation of the frequency of Th1 cells relative to the distance to the nearest blood vessel in the pUR4 or III-11C-treated dermis day 3-post OVA/CFA immunization. Statistics by two-way ANOVA, *p < 0.05, with Sidak's multiple comparisons **p < 0.01. Two to three independent experiments.

Given our unexpected results of the effect of pUR4 on tethering Th1 cells and attenuating migration *in vitro*, we re-visited the accumulation and position of Th1 cells *in vivo* in the inflamed dermis. IV-MPM images showed a significant accumulation of Th1 cells proximal to the pUR4 injection site that was not observed at the injection site of the control III-11C polypeptide, representative images (Figure 5A) and quantification (Figure 5B). To quantify Th1 accumulation, we harvested cells from the ear tissue and used flow cytometry to detect the OVA-specific Th1 cells within the ear tissue using a Thy1 mismatch between the host (Thy1.2^+^) and donor (adoptively transferred Thy1.1^+^) Th1 cells (Figure S4). We found a marked increase in both the frequency and number of donor Th1 cells in the pUR4-treated ears compared to control III-11C treatment (Figures 5C,D). Notably, the magnitude of Th1 accumulation at the pUR4 injection site, at least 6-fold higher than seen in other areas of the tissue (Figure 5B), was not reflected in the overall increase in Th1 cells in the tissue as measured by flow cytometry (a more modest 2-fold increase, Figure 5D and was not consistently marked by increases in other immune subsets such as myeloid cells to the tissue (data not shown). Therefore, it appears that the effect of pUR4 treatment is to primarily alter the relative positioning of Th1 cells within the tissue, rather than enhance tissue recruitment of immune cells.

To further examine the positioning of Th1 cells in the pUR4-treated dermis, the vasculature was labeled by injecting (i.v.) a fluorescently-conjugated CD31 Ab immediately prior to imaging (Figure 5E). 3D surface reconstruction of vessels using Imaris software (Figure 5E, lower panels), enabled calculation of the distance between each Th1 cell and the nearest blood vessel (Figure 5F). Th1 cells that accumulated in the pUR4-treated dermis were not observed within the blood vessels but were seen extra-vascularly in close proximity to blood vessels: coincident (0 μm) or less than one cell diameter (1–10 μm) from the vessel surface (Figure 5F). A significantly higher proportion of Th1 cells were located coincident with the blood vessels in the pUR4-treated dermis than in the control-treated dermis (Figure 5F). These data indicate that Th1 cell extravasation from the blood is not impaired by intradermal administration of pUR4, but ~40–50% of the Th1 cells that enter the tissue fail to migrate away from the vessels. Fibronectin is known to alter the assembly of collagen fibers and to modulate vascular endothelium, therefore we analyzed possible indirect effects of pUR4 treatment on the local milieu by assessing changes to the density of the collagen network and CD31^+^ vasculature. We found no alteration in SHG (as a surrogate for fibrillar collagen) or vessel density at the sites of Th1 accumulation (Figure S5). Thus, accumulation of Th1 cells in the dermis following pUR4 treatment, together with *in vitro* evidence for pUR4-mediated tethering (Figure 4), suggests that these cells get “stuck” peri-vascularly following pUR4-treatment.

### FN Manipulation Exacerbates Th1 Function in the Inflamed Dermis

**Figure 6:**
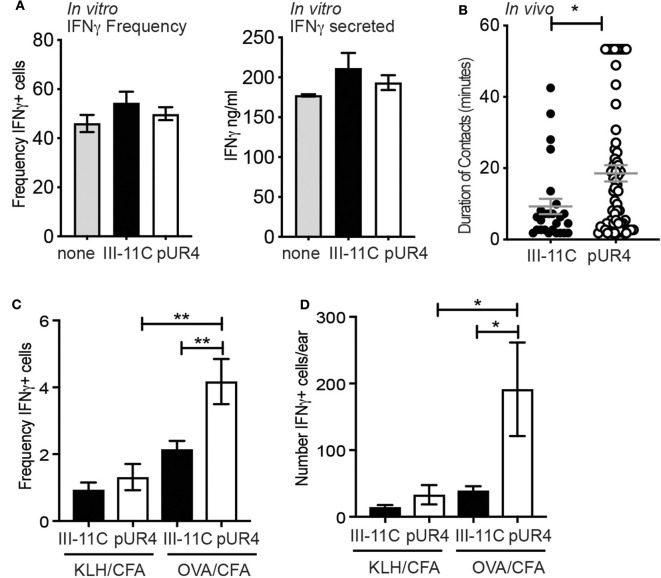
Enhanced Th1 function in the pUR4 treated inflamed dermis. **(A)** Naïve OVA-specific T cells stimulated *in vitro* with OVA-peptide, APC and pUR4 or III-11C polypeptides, under Th1-polarizing conditions. T cells were re-stimulated day 5 of culture with plate-bound anti-TCRβ Ab (H57) and the frequency of IFNγ-producers determined by intracellular cytokine staining and flow cytometry (left panel) and the amount of IFNγ secreted determined by ELISA (right panel). **(B)** Adoptively transferred Th1 cells were imaged by IV-MPM in the pUR4 or III-11C-treated dermis day 3-post OVA/CFA immunization. Duration of Th1 contacts with CD11c^+^ APCs was analyzed using Imaris surface tool to create 3D surfaces of T cells and APC. 3D volumetric overlap between the two cell surfaces was identified as a contact and measured over time to quantify the duration of T:APC cell contacts. Each symbol is an individual T:APC contact. Statistics by Mann Whitney, *p < 0.05. **(C,D)** OVA-specific Th1 cells were adoptively transferred to recipient mice that were treated with pUR4 or III-11C and immunized in one ear with cognate antigen, OVA/CFA, and in the contralateral ear with non-cognate antigen, KLH/CFA. Flow cytometric analysis of *ex vivo* IFNγ producing cells within the transferred Th1 cells. **(C)** Frequency and **(D)** number of IFNγ producing cells by the transferred Th1 cells in the presence (OVA) and absence (KLH) of cognate antigen in the pUR4 or III-11C treated inflamed ear. Statistics were performed using one-way ANOVA with Tukey's multiple comparisons test. **p < 0.01, *p < 0.05. Two to three independent experiments, 4–5 mice per group per experiment.

To determine the functional impact of perivascular Th1 cell accumulation following pUR4 treatment, we assessed the effects of pUR4 treatment on T cell activation. FN has been implicated in the co-stimulation of T cells (48) and therefore pUR4 may have direct modulatory effects on T cell activation independent of effects on FN. To test this, naïve DO11.10 TCR Tg^+^ T cells were activated *in vitro* with OVA-peptide and APC under Th1 polarizing conditions in the presence of pUR4 or control III-11C. After 5 days, the T cells were re-stimulated and effector function determined by measuring the canonical Th1 cytokine, IFNγ. The frequency of IFNγ-producing cells was assessed by intracellular cytokine staining and flow cytometry, and by the secretion of IFNγ by ELISA (Figure 6A). Treatment of T cell activation cultures with pUR4 had no effect on the generation or function of Th1 cells (Figure 6A). Therefore, pUR4 does not appear to directly alter T cell activation.

T cell activation was next determined *in vivo*, measured by assessing the duration of T:APC interactions and the *in situ* secretion of IFNγ by *ex-vivo* cytokine staining (43). Fluorescently-labeled OVA-specific Th1 cells were transferred to pUR4-treated and OVA/CFA immunized mice (as in Figure 1) and T:APC interactions were measured in real time with IV-MPM by acute labeling of CD11c^+^ APC with anti-CD11c-PE Ab i.d. injected 2 h prior to imaging. The duration of Th1 cell interactions with CD11c^+^ cells was measured using an unbiased automated 3D surface rendering tool in Imaris (49) (Figure S6). Th1 cells had significantly longer interaction times with APCs in the pUR4-treated inflamed dermis than the control group (Videos 6, 7), with many Th1s remaining in contact with APCs for the 50–60 min imaging period (Figure 6B) (Video 6). To determine if prolonged APC contact enhanced effector function, we measured *ex-vivo* expression of IFNγ by intracellular cytokine staining and flow cytometry. OVA-specific Th1 cells were transferred to mice immunized with OVA/CFA (cognate antigen) in one ear and KLH/CFA (non-cognate antigen) in the contralateral ear. On day 3 post-immunization, cells were harvested from the inflamed ears in Brefeldin-A-containing buffers to directly assess *ex-vivo* cytokine production (43). The frequency and number of IFNγ^+^ cells were significantly increased in the pUR4-treated ears in an antigen-specific manner (Figures 6C,D). pUR4-treatment did not result in non-specific T cell activation as there was no increase in the number of IFNγ producers in pUR4-treated ears immunized with the non-cognate antigen, KLH (KLH/CFA). Thus, pUR4-treatment of the inflamed dermis confined the movement of Th1 cells leading to longer T:APC contacts and enhanced cytokine production, unexpectedly exacerbating inflammation.

## Discussion

The movement of T cells within infected tissues is critical for effective pathogen clearance and tissue repair, yet the T cell guidance cues used to navigate inflamed tissues are poorly understood. Our previous work using IV-MPM had revealed that Th1 cells utilize the ECM as a scaffold for integrin-dependent migration (9). We now show that FN is a critical player in facilitating such T cell interstitial migration. The use of fluorescently-tagged FN as a real-time probe for FN *in situ* enabled the first *in vivo* visualization of T cell migration along FN fibers, revealing an active interplay between migrating T cells and the flexible FN scaffold. Utilizing a polypeptide derived from a bacterial adhesion that blocks FN deposition (29), pUR4, we were able to locally manipulate FN matrix assembly. Disrupting FN deposition in the inflamed dermis led to a marked inhibition of T cell migration. We identify two mechanisms of pUR4 action: inhibiting T cell migration via limiting substrate availability and/or inhibiting migration due to enhancing substrate adhesion thus tethering cells in place. The functional consequence of pUR4-treatment was to unexpectedly exacerbate T cell accumulation at the inflamed site and enhance the inflammatory cytokine IFNγ production.

Real time imaging of T cells and the ECM has relied on the multiphoton microscopy-generated SHG signal as a surrogate for a fibrillar scaffold but does not reflect the actual substrate along which the T cells migrate. Using fluorescent FN molecules incorporated into nascent FN fibrils we were able to visualize the interface between migrating T cells and their ECM substrate. FN associated with fibers coincident with SHG (type III collagen by IHC), and also formed distinct fibers not associated with SHG. Using this intra-vital approach, the flexibility of the FN fibers within the inflamed dermis was clearly visible, with fibers being temporarily deformed as the Th1 cells migrated (Video 1). Such flexibility is intriguing given the growing interest in mechano-sensing mechanisms in immunity (50, 51) and in how substrate stiffness can influence ECM assembly and function of interacting cells (52, 53). FN and collagen assembly is a cooperative process (54), with *in vitro* studies showing that collagen preferentially colocalizes with more relaxed FN (55). In turn, excess FN matrix deposition (56) or tension placed on FN fibers (57), and now pUR4-alteration in FN deposition as we reveal here, attenuates the rate of cell migration; with enhanced stress decreasing cell migration possibly through a conformation change in FN that enhances integrin-binding (57). A recent study using tunable biomaterials revealed that migrating cells utilize the flexibility of the matrix to enhance migration (58). Cell contractility led to matrix stretch and recoil resulting in a rapid migration mode that they termed “sling shot migration” (58). The ability to now assess, *in vivo*, the flexibility of FN fibers relative to migrating immune cells will facilitate analysis of the relationship between substrate flexibility, migratory preference and speed as cells navigate inflamed sites.

Mimetics of bacterial adhesins that bind FN and prevent its assembly into fibrils represent an exciting approach to attenuate fibrotic disease. Systemic delivery of pUR4/FUD has been shown to significantly reduce FN deposition, decrease innate immune infiltration and attenuate cardiac and liver fibrosis in mouse models (23–25). These models of chronic disease are often associated with the recruitment and activation of macrophages and it will be interesting to determine if there is a difference in the effect of FN blockade between different immune cell types. Delivery of pUR4 peri-adventitially in a model of vascular remodeling resulted in a marked decrease in leukocyte infiltration into the vessel wall that correlated with decreased vessel expression of ICAM-1 and VCAM-1 (23), adhesion molecules critical for immune cell extravasation (2). Our approach to administer pUR4 intradermally would bypass any action of FN blockade on vascular control of immune cell extravasation. However, the previously observed pUR4-induced changes in adhesion molecules on blood vessels (23), raises the possibility that a similar alteration in adhesion molecules might occur on tissue lymphatics (59), if pUR4 is administered intradermally. Therefore, it will be interesting to determine if T cell accumulation following pUR4 treatment could be in part explained by reduced exit from the tissue via lymphatics.

Our studies suggest that pUR4 may act to enhance cellular adhesion, possibly through changing the conformation of FN. *In vitro* studies have shown that binding of pUR4/FUD to soluble FN leads to conformational changes that “expand” the protein to expose the FNIII module that contains the RGD sequence for integrin binding (31, 32). Structural studies with a related *S. aureus* FN-binding protein demonstrated that the conformational rearrangement of soluble FN enhanced FN/α5β1 integrin affinity as measured by surface plasmon resonance (33). Our own *in vitro* studies support this notion, with T cells appearing tethered to the pUR4-treated FN surface (Video 5). *In vivo*, the T cells also appeared “stuck” perivascularly, with cells spreading as if strongly adhered. We had predicted that in the absence of being able to effectively scan the tissue to encounter APCs for activation, T cells would have reduced effector function, but instead perivascular accumulation enhanced T function, perhaps linked to the co-localized perivascular clustering of APCs implicated in boosting T cell activation (60). ECM fragments have been implicated in directly activating T cells (61–63). However, we found no direct evidence of pUR4 acting directly on the T cells themselves. Rather, it appears that the lack of ability of T cells to move away from antigen-bearing APC in the pUR4-treated microenvironment may prolong activation times and exacerbate cytokine production. Therefore, we show here that too much integrin-based adhesion limits T cell locomotion and have shown previously that limiting integrin-based adhesion, by acute blockade of T cell α_V_-integrin association with the matrix (9), also results in an attenuation of T cell locomotion. These experimental results fit well with a proposed model of migration efficiency that tunes T cell migration by balancing the degree of adhesion, too much or too little adhesion leading to cellular arrest (47). Interestingly, we find that limiting adhesion in different ways leads to distinctly different functional outcomes: the tethering effect of pUR4 enhances T:APC interactions and IFNγ production while blocking interstitial migration with acute anti-α_V_ Ab treatment decreased IFNγ production presumably by limiting the ability of the Th1 cells to “find” APC (9). Future therapeutic strategies that target adhesion may therefore have distinct (and opposite) functional outcomes depending on the spatiotemporal administration of the therapeutic.

Our findings on the use of pUR4-treatment for targeted inhibition of FN deposition highlight an important context-dependent effect on T cell mediated immunity. Instead of dampening inflammation, pUR4 delivered locally within a tissue enhanced T cell accumulation. Our results raise the interesting possibility that FN targeting through pUR4 treatment may be useful in enhancing T cell accumulation and activation at sites of chronic infection or in regions of the tumor that would otherwise be inaccessible.

## Data Availability Statement

The raw data supporting the conclusions of this article will be made available by the authors, without undue reservation.

## Ethics Statement

The animal study was reviewed and approved by University of Rochester's Institutional Animal Care and Use Committee.

## Author Contributions

NF designed, performed, and analyzed most of the experiments with assistance from DS and NR. NR and PO designed and performed the *in vitro* migration experiments. DH provided critical reagents and helped with conceptual and experimental design. DF conceived and supervised the project. NF and DF wrote the manuscript. All authors contributed to the article and approved the submitted version.

## Conflict of Interest

The authors declare that the research was conducted in the absence of any commercial or financial relationships that could be construed as a potential conflict of interest.

## Supplementary Material

Video 1 Th1 cells migrating along fibronectin fibers in the CFA-inflamed dermis. OVA-specific Th1 cells labeled with CMTMR (blue) migrating along AF-488 conjugated FN (green) fibers in the OVA/CFA-inflamed dermis on d3 of inflammation. Cells were imaged using IV-MPM for 30 min. Th1 cells appear to be pushing and pulling along flexible fibronectin fibers.

Videos 2,3 Decreased Th1 cell interstitial motility proximal to the pUR4 injection site. Adoptively transferred OVA-specific Th1 cells (green) and Second Harmonic Generation (SHG) (blue) in the pUR4 (Video 2) or III-11c (Video 3) treated OVA/CFA-inflamed dermis were captured for 30 min. Side-by-side video proximal and distal from the injection site using IV-MPM imaging. Scale Bar: 100 μm. Enhanced accumulation and decreased Th1 cell motility local to the pUR4 injection site in the CFA-inflamed dermis.

Video 4 pUR4 T cell pre-treatment does not have a direct effect of CD4 Th1 cell migration *in vitro*. Th1 cells (yellow) were pretreated with 500 nM III-11c (left) or pUR4 (right) and imaged under agar on fibronectin-coated glass coverslips using confocal microscopy. Twenty minutes movies of Th1 cell migration. Scale Bar: 100 μm.

Video 5 Fibronectin pretreated with pUR4 alters CD4 Th1 cell migration *in vitro*. Fibronectin was pretreated with 500 nM III-11c (left) or pUR4 (right) and then coated on glass coverslips. Twenty minutes movies of Th1 cells (yellow) migrating under agar on glass coverslips coated with pre-treated fibronectin were captured using confocal microscopy. Scale Bar: 100 μm.

Video 6 Prolonged T:APC interactions at the pUR4-treated site. Fluorescently labeled Th1 cells were transferred into OVA/CFA immunized mice with dermal pUR4 treatment (as described in Figure 2) and imaged 3 days later. Mice were injected intradermally with anti-CD11c-PE antibody and Fc-block 2 h prior to imaging. The video shows a representative prolonged T cell (green) encounter with CD11c^+^ cells (red). Th1 and CD11c surfaces were rendered using the Imaris (Bitplane) surface tool, and dynamic cell surface overlap (white) used to determine unbiased T:APC contact time (see Figure 6, Figure S6).

Video 7 Duration of T:APC interactions at the III-11c-treated site. Fluorescently labeled Th1 cells (green) were transferred into OVA/CFA immunized mice with dermal III-11c treatment (as described in Figure 2) and imaged 3 days later. Mice were injected intradermally with anti-CD11c-PE antibody and Fc-block 2 h prior to imaging. The video shows representative multiple T cell (green) encounters with CD11c^+^ cells (red). Th1 and CD11c surfaces were rendered using the Imaris (Bitplane) surface tool, and dynamic cell surface overlap (white) used to determine unbiased T:APC contact time (see Figure 6, Figure S6).

Figure S1 Association with fibronectin fibers in SHG and T cells in the CFA-inflamed dermis. **(A,B)** WT mice were immunized with OVA/CFA in the ear pinna. On d3 of inflammation. Hundred microgram FN-AF488 was i.v. injected 4 h prior to IV-MPM imaging. **(A)** An example of a single Z-plane from a MP image stack showing the local orientation overlays of the fibronectin (yellow) and collagen (SHG) (red) signals. **(B)** Histograms of the orientation angles of the fibronectin and collagen (SHG) and random distributions for the images in **(A)**. **(C,D)** OVA-specific Th1 cells labeled with CMTMR (red) were adoptively transferred to WT mice and immunized with OVA/CFA in the ear pinna. On d3 of inflammation, 100 μg FN-AF488 was i.v. injected 4 h prior to IV-MPM imaging. SHG (gray), AF-488 FN (green) and Th1 cells (red) of a 2D slice **(C)** in a 3D stack **(D)** in the OVA/CFA-inflamed dermis, scale bar 50 and 100 μm respectively. Large red structures surrounded by areas devoid of SHG are hair follicles with auto fluorescent hair fibers.

Figure S2 FN deposition on glass coverslips. Two hundred nanometer FN was pre-incubated with 500 nM pUR4 or III-11C or left untreated (No Tx) for 30 min at RT before coating the coverslip for 60 min at RT. The coverslip was stained with rabbit polyclonal anti-FN Ab followed by Alexa Fluor 488 (AF-488)-conjugated donkey anti-rabbit Ab and visualized using confocal microscopy. Mean fibronectin pixel intensity was quantified using FIJI software.

Figure S3 Maximum projection images of T cell migratory paths on III-11C or pUR4 pre-treated FN. Twenty minutes movies of Th1 cells migrating under agar on glass coverslips coated with pUR4 or III-11C pre-treated FN were captured using confocal microscopy. Binary images were produced using a triangle thresholding algorithm in Python. Tracks were generated using TrackPy in Python. Cell tracks from each frame in the time lapse were added together and displayed on a single image.

Figure S4 Gating strategy for detection of transferred Th1 cells. 7.5 × 10^6^ OVA-specific Thy 1.1^+^ Th1 cells were adoptively transferred into WT Thy 1.2^+^ mice and treated with pUR4 or III-11C and immunized as described in Figure 2. Cells were harvested from the ear tissue, labeled with live/dead stain, anti-CD45, anti-CD4 and anti-Thy1.1 Abs and analyzed using flow cytometry. Gating sequence: Live, FSC; singlets; CD45+; CD4+; Thy1.1, CD4. The adoptively transferred cells (donor) were distinguished from endogenous CD4 T cells (host) by expression of Thy1.1.

Figure S5 pUR4 treatment does not alter the local collagen (SHG) or vessel density. Mice were injected intradermally with pUR4 or control III-11C and immunized with OVA/CFA as described in Figure 2. On d3 of inflammation, the density of SHG and CD31+ vessels was calculated in areas proximal or distal to the injections site. **(A)** Representative multiphoton images of the SHG signal proximal to the injection site. **(B)** Density of SHG measured by calculating the percentage of the planar area filled by SHG signal. **(C)** SHG density in individual imaging fields relative to the injection site. **(D)** Density of CD31+ vessels proximal to the injection site measured by calculating the percentage of the planar area filled by CD31 fluorescence (see images in Figure 5). **(A–D)** All data not statistically significant. Representative data from 2 to 3 independent experiments, 2–3 mice per experiment, 2–3 imaging fields per mouse.

Figure S6 Real time T:APC contact analysis by IV-MPM. OVA-specific Th1 cells (green) were adoptively transferred into WT mice treated with pUR4 or III-11C and immunized as described in Figure 2. CD11c+ APCs (red) were detected by intradermal injection of a mixture of 1μg CD11c-PE and Fc Block (CD16/32) Abs 2 h prior to IV-MPM imaging. For T:APC contact analysis, 3D masked surfaces for CD11c+ and T cells were generated in Imaris and the 3D volumetric overlap between the two cell surfaces identified as a contact (white) and the duration of individual contacts determined over time. **(A,B)** time lapse sequences of two examples of individual T:APC contacts in the inflamed dermis.
