# Supplementary figures and images for: CD4+ T Cell Interstitial Migration Controlled by Fibronectin in the Inflamed Skin
Source: Front Immunol. 2020 Jul 24;11:1501. doi: 10.3389/fimmu.2020.01501 (PMC7393769; doi:10.3389/fimmu.2020.01501)

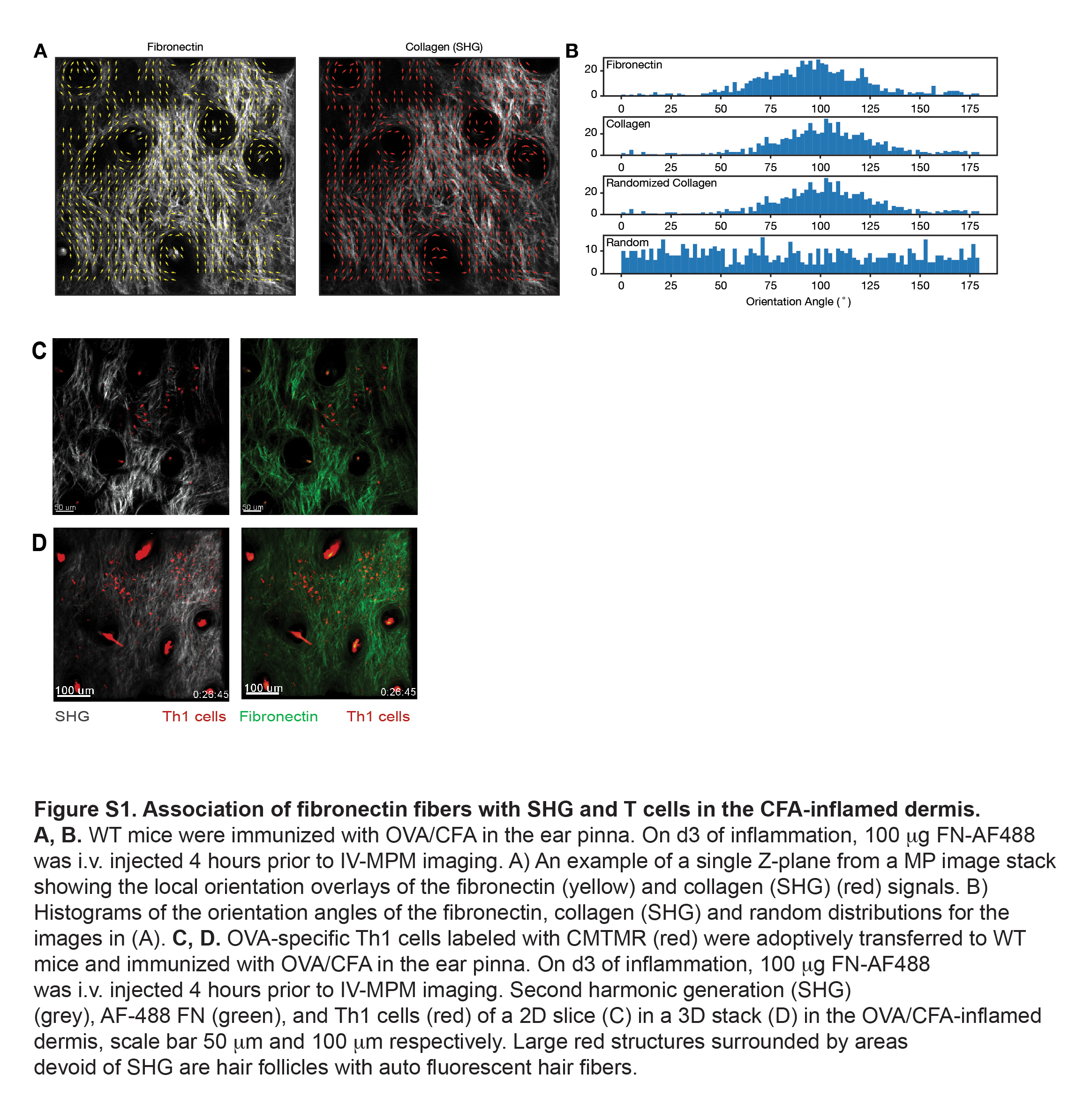

Supplement: Figure S1 — Association with fibronectin fibers in SHG and T cells in the CFA-inflamed dermis. (A,B) WT mice were immunized with OVA/CFA in the ear pinna. On d3 of inflammation. Hundred microgram FN-AF488 was i.v. injected 4 h prior to IV-MPM imaging. (A) An example of a single Z-plane from a MP image stack showing the local orientation overlays of the fibronectin (yellow) and collagen (SHG) (red) signals. (B) Histograms of the orientation angles of the fibronectin and collagen (SHG) and random distributions for the images in (A). (C,D) OVA-specific Th1 cells labeled with CMTMR (red) were adoptively transferred to WT mice and immunized with OVA/CFA in the ear pinna. On d3 of inflammation, 100 μg FN-AF488 was i.v. injected 4 h prior to IV-MPM imaging. SHG (gray), AF-488 FN (green) and Th1 cells (red) of a 2D slice (C) in a 3D stack (D) in the OVA/CFA-inflamed dermis, scale bar 50 and 100 μm respectively. Large red structures surrounded by areas devoid of SHG are hair follicles with auto fluorescent hair fibers. [file Image_1.JPEG]

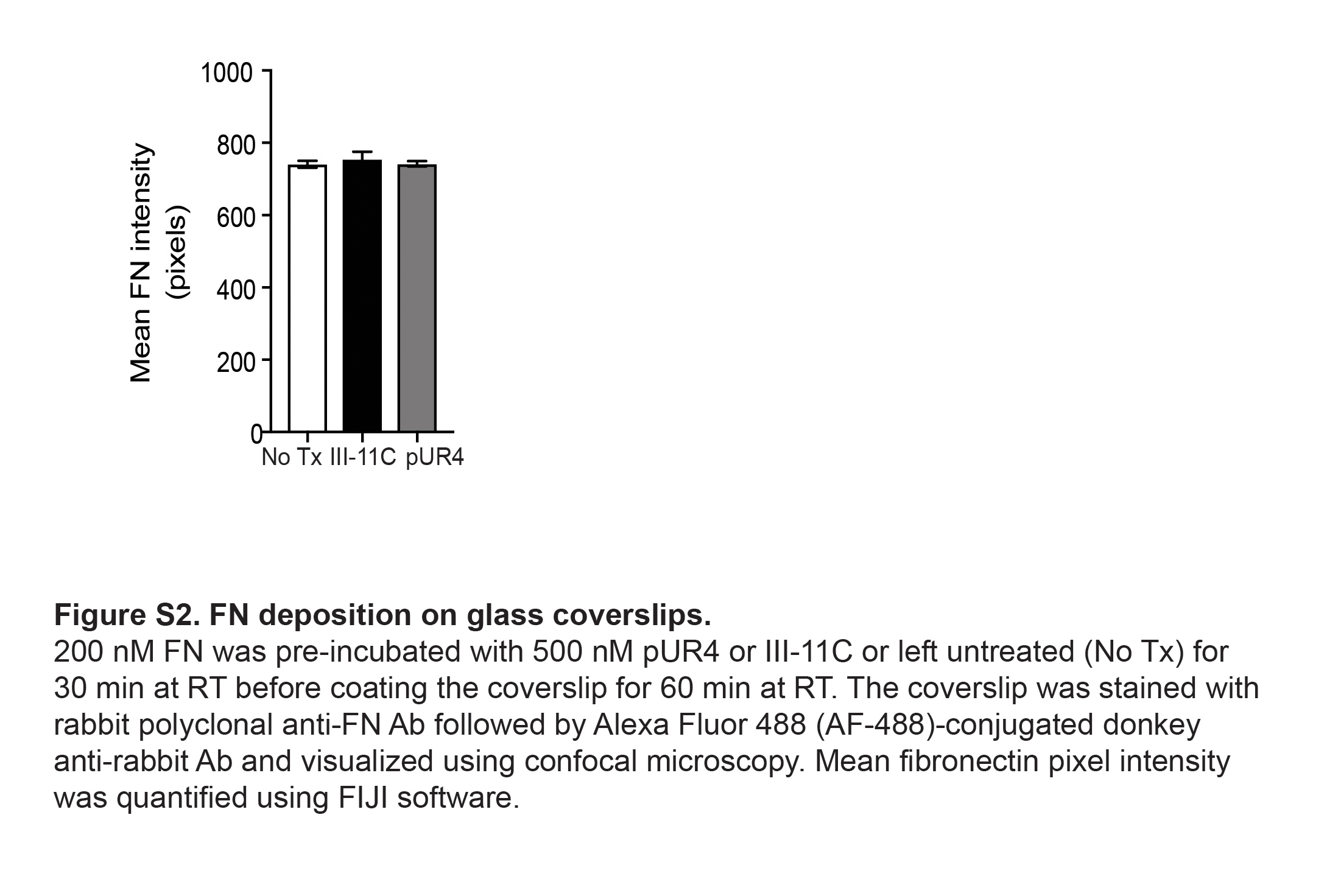

Supplement: Figure S2 — FN deposition on glass coverslips. Two hundred nanometer FN was pre-incubated with 500 nM pUR4 or III-11C or left untreated (No Tx) for 30 min at RT before coating the coverslip for 60 min at RT. The coverslip was stained with rabbit polyclonal anti-FN Ab followed by Alexa Fluor 488 (AF-488)-conjugated donkey anti-rabbit Ab and visualized using confocal microscopy. Mean fibronectin pixel intensity was quantified using FIJI software. [file Image_2.JPEG]

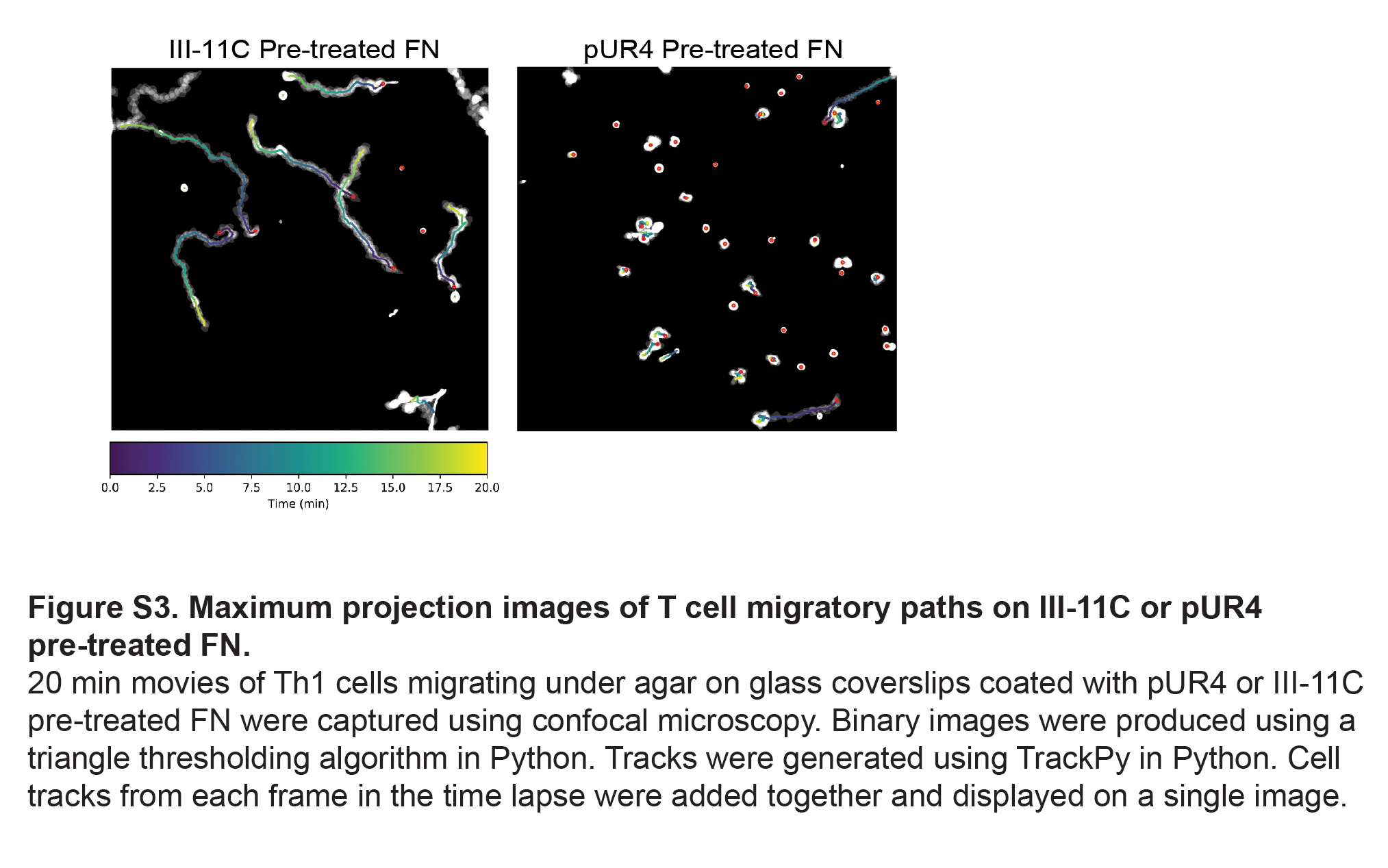

Supplement: Figure S3 — Maximum projection images of T cell migratory paths on III-11C or pUR4 pre-treated FN. Twenty minutes movies of Th1 cells migrating under agar on glass coverslips coated with pUR4 or III-11C pre-treated FN were captured using confocal microscopy. Binary images were produced using a triangle thresholding algorithm in Python. Tracks were generated using TrackPy in Python. Cell tracks from each frame in the time lapse were added together and displayed on a single image. [file Image_3.JPEG]

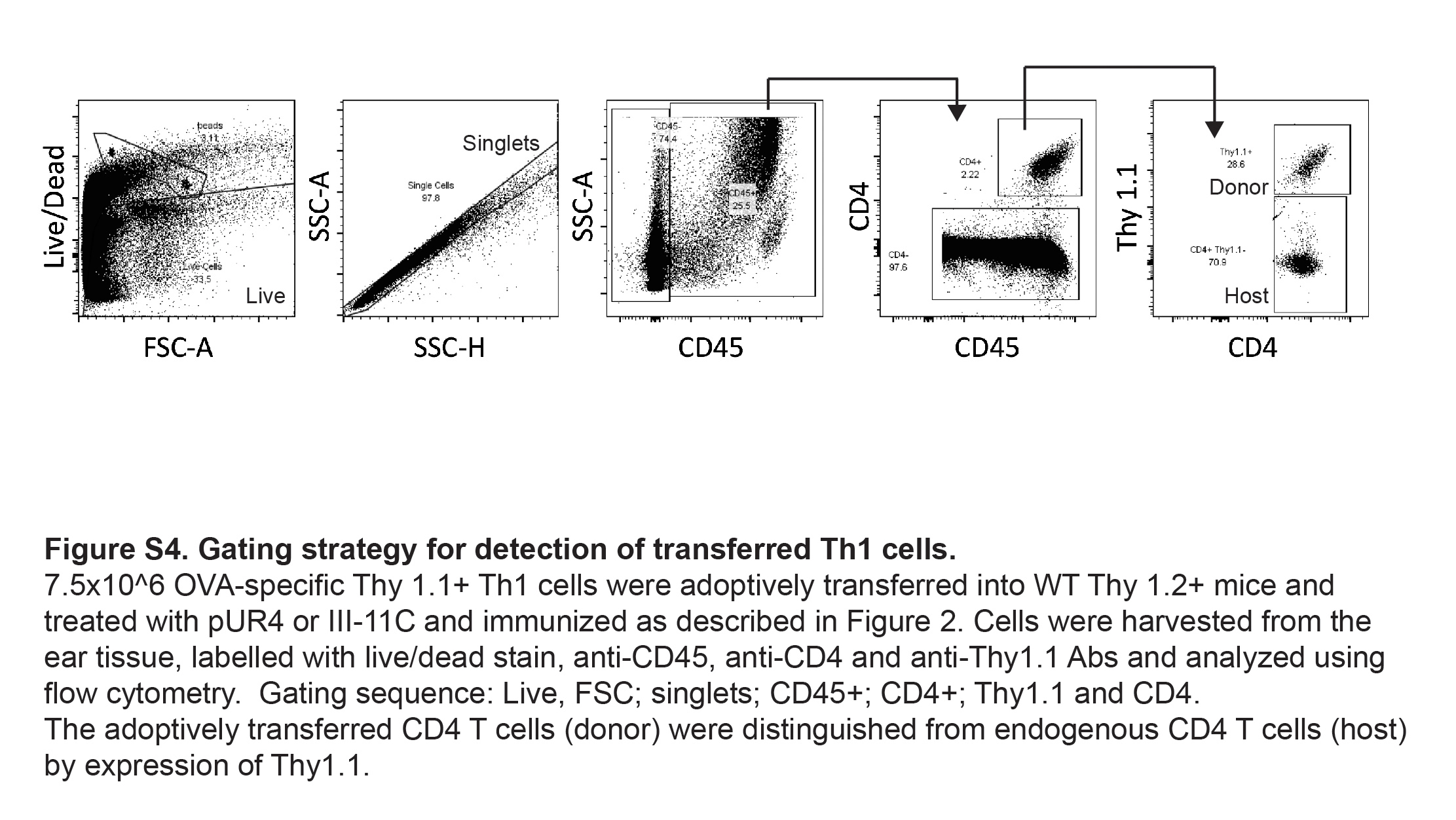

Supplement: Figure S4 — Gating strategy for detection of transferred Th1 cells. 7.5 × 10^6^ OVA-specific Thy 1.1+ Th1 cells were adoptively transferred into WT Thy 1.2+ mice and treated with pUR4 or III-11C and immunized as described in Figure 2. Cells were harvested from the ear tissue, labeled with live/dead stain, anti-CD45, anti-CD4 and anti-Thy1.1 Abs and analyzed using flow cytometry. Gating sequence: Live, FSC; singlets; CD45+; CD4+; Thy1.1, CD4. The adoptively transferred cells (donor) were distinguished from endogenous CD4 T cells (host) by expression of Thy1.1. [file Image_4.JPEG]

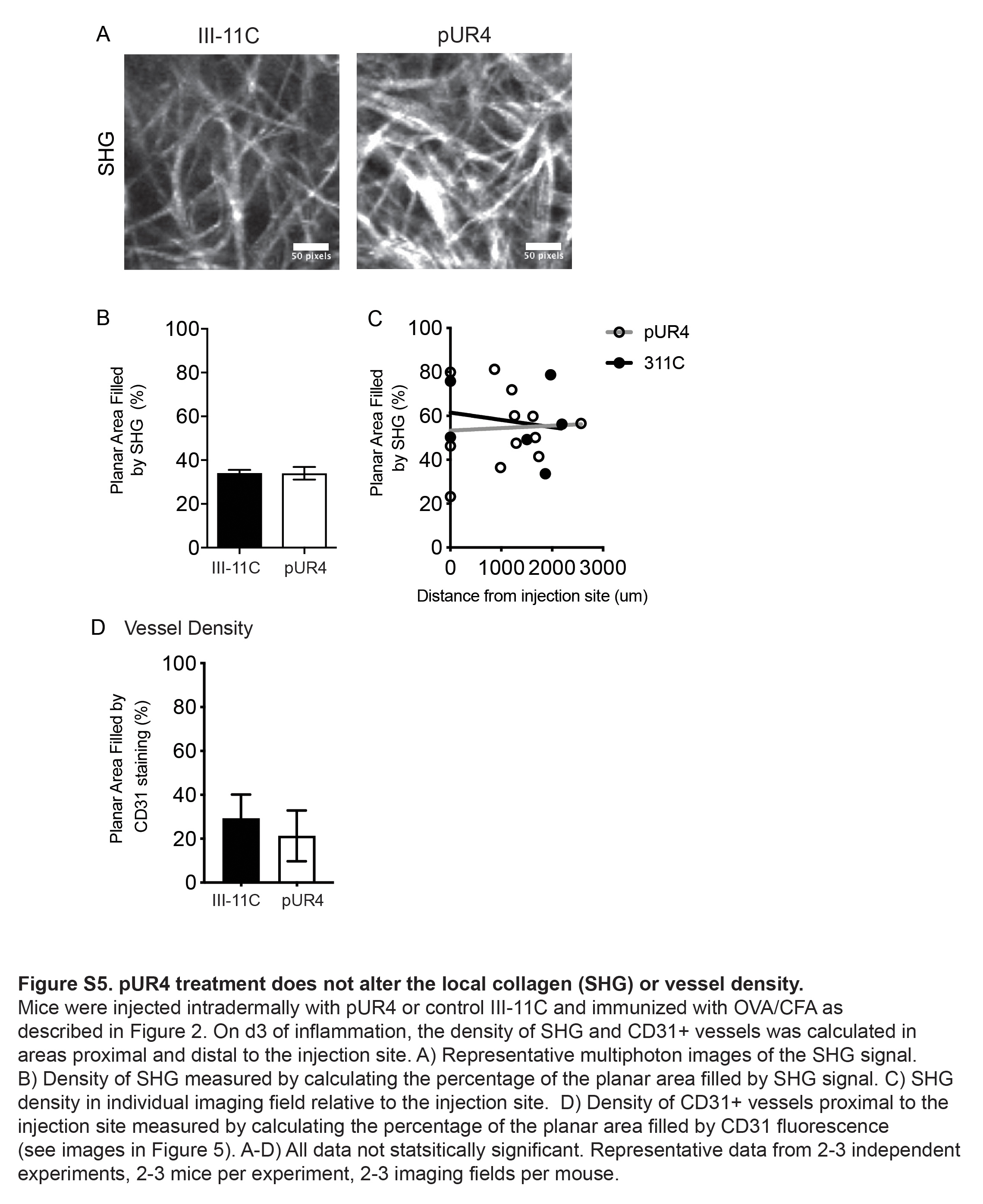

Supplement: Figure S5 — pUR4 treatment does not alter the local collagen (SHG) or vessel density. Mice were injected intradermally with pUR4 or control III-11C and immunized with OVA/CFA as described in Figure 2. On d3 of inflammation, the density of SHG and CD31+ vessels was calculated in areas proximal or distal to the injections site. (A) Representative multiphoton images of the SHG signal proximal to the injection site. (B) Density of SHG measured by calculating the percentage of the planar area filled by SHG signal. (C) SHG density in individual imaging fields relative to the injection site. (D) Density of CD31+ vessels proximal to the injection site measured by calculating the percentage of the planar area filled by CD31 fluorescence (see images in Figure 5). (A–D) All data not statistically significant. Representative data from 2 to 3 independent experiments, 2–3 mice per experiment, 2–3 imaging fields per mouse. [file Image_5.JPEG]

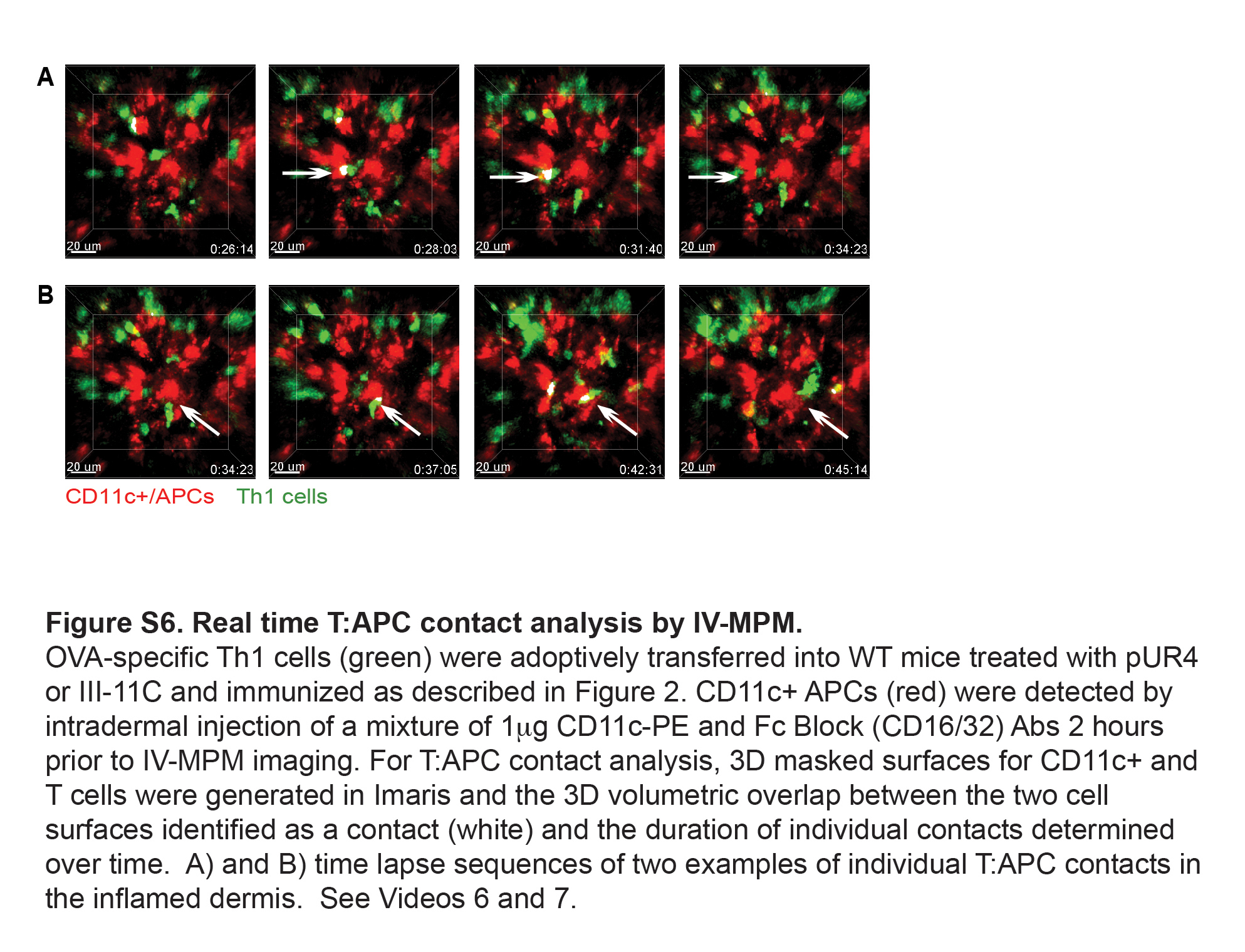

Supplement: Figure S6 — Real time T:APC contact analysis by IV-MPM. OVA-specific Th1 cells (green) were adoptively transferred into WT mice treated with pUR4 or III-11C and immunized as described in Figure 2. CD11c+ APCs (red) were detected by intradermal injection of a mixture of 1μg CD11c-PE and Fc Block (CD16/32) Abs 2 h prior to IV-MPM imaging. For T:APC contact analysis, 3D masked surfaces for CD11c+ and T cells were generated in Imaris and the 3D volumetric overlap between the two cell surfaces identified as a contact (white) and the duration of individual contacts determined over time. (A,B) time lapse sequences of two examples of individual T:APC contacts in the inflamed dermis. [file Image_6.JPEG]
